# GILT Expression in Human Melanoma Cells Enhances Generation of Antigenic Peptides for HLA Class II-Mediated Immune Recognition

**DOI:** 10.3390/ijms23031066

**Published:** 2022-01-19

**Authors:** Jessica D. Hathaway-Schrader, Duncan Norton, Katherine Hastings, Bently P. Doonan, Shaun Tompkins Fritz, Jennifer R. Bethard, Janice S. Blum, Azizul Haque

**Affiliations:** 1 Department of Microbiology and Immunology, Hollings Cancer Center, Children’s Research Institute, Medical University of South Carolina, 173 Ashley Avenue, Charleston, SC 29425, USA; hathawa@musc.edu (J.D.H.-S.); duncan.norton@prismahealth.org (D.N.); hastingskra@gmail.com (K.H.); bently.doonan@medicine.ufl.edu (B.P.D.); shauntompkins@gmail.com (S.T.F.); 2 UF Brain Tumor Immunotherapy Program, Department of Oncology, University of Florida, Gainesville, FL 3260, USA; 3 Department of Cell and Molecular Pharmacology and Experimental Therapeutics, Medical University of South Carolina, 173 Ashley Avenue, Charleston, SC 29425, USA; bethard@musc.edu; 4 Department of Microbiology and Immunology, School of Medicine, Indiana University, Indianapolis, IN 46202, USA; jblum@iupui.edu

**Keywords:** gamma-IFN-inducible lysosomal thiol-reductase (GILT), melanoma, Ag presenting cells (APCs), human leukocyte antigen (HLA) class II, cathepsins, CD4+ T cells

## Abstract

Melanoma is an aggressive skin cancer that has become increasingly prevalent in western populations. Current treatments such as surgery, chemotherapy, and high-dose radiation have had limited success, often failing to treat late stage, metastatic melanoma. Alternative strategies such as immunotherapies have been successful in treating a small percentage of patients with metastatic disease, although these treatments to date have not been proven to enhance overall survival. Several melanoma antigens (Ags) proposed as targets for immunotherapeutics include tyrosinase, NY-ESO-1, gp-100, and Mart-1, all of which contain both human leukocyte antigen (HLA) class I and class II-restricted epitopes necessary for immune recognition. We have previously shown that an enzyme, gamma-IFN-inducible lysosomal thiol-reductase (GILT), is abundantly expressed in professional Ag presenting cells (APCs), but absent or expressed at greatly reduced levels in many human melanomas. In the current study, we report that increased GILT expression generates a greater pool of antigenic peptides in melanoma cells for enhanced CD4+ T cell recognition. Our results suggest that the induction of GILT in human melanoma cells could aid in the development of a novel whole-cell vaccine for the enhancement of immune recognition of metastatic melanoma.

## 1. Introduction

Melanoma is one of the most rapidly growing cancers plaguing western populations [1,2,3]. In 2021, an estimated 106,110 new cases of invasive melanoma will be diagnosed in the US (https://www.cancer.org/research/cancer-facts-statistics/all-cancer-facts-figures/cancer-facts-figures-2020.html; accessed on 30 December 2021), and an estimated 7180 people will die of melanoma [4]. Treatments of early stage melanoma include surgery, radiation, and chemotherapy, which are generally effective at eradicating the disease; but in the case of late stage metastatic melanoma, there is no curative option [4,5,6]. To fill this gap in viable treatment options, immunotherapy has been tried in multiple forms over the past decade. Of these, autologous T cell transfer, interferon therapy, whole cell cancer vaccines, and antibody therapy (e.g., ipilimumab and pembrolizumab) have had some success [7,8,9], yet a broadly effective immunotherapy remains elusive. While combination therapies of checkpoint inhibitors ipilimumab and nivolumab confer a significant survival benefit in patients with metastatic melanoma [10], they come with some potentially serious side effects linked to inflammation. However, recent preclinical work has also highlighted the non-overlapping role of neoantigen responses mediated by CD4+ and CD8+ T cells [11]. One of the main failures associated with melanoma immunotherapy is reduced stimulation of both CD4+ and CD8+ T cells [12,13], leading to incomplete tumor clearance and immunological memory. A recent study showed that the downregulation of GILT by lentiviral-mediated silencing inhibited cell growth, colony formation, and migration. The inhibition of GILT also promoted apoptosis and reduced tumor growth in vivo [14]. Although GILT expression in professional APCs enhances MHC class II-restricted presentation [15,16,17], a recent study also showed that GILT expression in thymic APCs and medullary thymic epithelial cells may increase Treg cells and could diminish antitumor response to a tissue-restricted, melanoma-associated self-antigen [18]. While our group could not find any differences in MHC class I presentation upon GILT expression [15], a recent study has shown that GILT is also able to facilitate anti-tumor immune surveillance via promoting MHC class I mediated-antigen presentation in colon carcinoma [19]. This study in a murine model found that GILT expression could also reduce tumor growth in vivo with the infiltration of leucocytes, but had no effect on tumor cell development. Our current study looks to dissect novel antigen (Ag) processing reactions in melanoma that could be exploited for loaded dendritic cell and melanoma whole-cell vaccine strategies. 

Melanoma Ags MART-1, NY-ESO-1, gp100, and tyrosinase are broadly expressed in human tumors [20,21,22,23,24,25,26]. These Ags have been shown to elicit both cytotoxic CD8+ and helper CD4+ T cell responses [12]. CD8+ T cells and natural killer cells play key roles in eliminating transformed or malignant cells [27]. While cytotoxic CD8+ T cells are able to destroy melanoma cells [28], their interaction with tumor cells and killing efficiency are greatly supported by CD4+ helper T cells, which produce inflammatory cytokines and chemokines to accelerate host immunity. Melanoma Ags expressed by tumors must be processed to short peptides, which then bind to human leukocyte antigen (HLA) class I and II molecules, with these resulting peptide–HLA complexes engaging T cells to promote their activation and killing functions. The capture of fragments of tumor cells by professional Ag-presenting cells (APC) including dendritic cells also results in the processing and HLA-restricted presentation of tumor peptides to T cells. Yet tumors have evolved multiple mechanisms of immune evasion, including loss of tumor Ag expression or post-translational modification of these Ags, which can mask tumor cells from recognition and T cell-induced cell death. The Ags MART-1, NY-ESO-1, gp100, and tyrosinase contain multiple epitope sequences, which would be predicted to readily bind to HLA class II molecules, yet many of which contain cysteine residues. Given their susceptibility to oxidation and post-translational modification, cysteine residues within an Ag can significantly impact whether an epitope binds to MHC class II molecules or promotes CD4+ T cell recognition [20,24,25,29]. The thiol reductase GILT has been shown to efficiently reduce cystine and other oxidized cysteine residues within proteins and peptides, to improve the binding of select epitopes to HLA class II molecules, as well as enhancing T cell recognition [15,30,31]. While GILT is expressed at high levels by activated dendritic cells [32,33], the expression of this reductase is highly variable in human melanomas and skin cells [32,34]. GILT expression in B lymphocytes also impacts the expression of lysosomal proteases, suggesting perhaps an even larger role for this reductase in tumor Ag processing and presentation. 

GILT expression can be induced in melanoma and other tumor cells by treatment with IFN-γ [17,18,35]. Within APCs, GILT is found in its proforms in early endosomes, while the mature form is found in multivesicular late endosomes and multilaminar lysosomes [17,36,37]. GILT is active at low pH and catalyzes the breaking of disulfide bonds, making Ags/peptides susceptible to degradation by acidic cathepsins. The role of GILT in Ag presentation may be complex, with evidence that this reductase impacts CD4+ and CD8+ T cell responses [15,16,32,38,39,40]. GILT appears to play a role in Ag cross-presentation by dendritic cells for epitopes recognized by CD8+ T cells [41], but in the case of CD4+ T cell responses to melanoma, cells as well as APC expressions of GILT may impact tumor clearance [15,32,38]. While much is known about the pathways for Ag processing in professional APCs, less is understood about the regulation of the HLA class II pathway for epitope presentation in melanomas. HLA class II proteins are synthesized in the endoplasmic reticulum (ER) of cells, which form a complex with the invariant chain (Ii) [42,43,44]. In the endolysosomal compartments, proteases degrade Ii, leaving class II-associated Ii peptide (CLIP) on the binding groove [45,46,47,48]. Ags are also processed by acidic proteases and GILT in the endocytic pathway [45,49,50], where the non-classical class II molecule, HLA-DM, chaperones the removal of CLIP, forming a stable class II–peptide complex for presentation to CD4+ T cells [51,52,53]. A recent study has also suggested the variable expression of GILT in tumor cells and infiltrating APCs. Understanding more precisely the molecular mechanisms by which GILT controls class II presentation in melanoma cells is therefore important and may offer novel insights for the development of more effective vaccines for melanoma and other solid tumors. 

## 2. Results

### 2.1. GILT Expression Influences Invariant Chain (Ii) and HLA-DM Protein Expression in Human Melanoma Cell Lines

**Figure 1:**
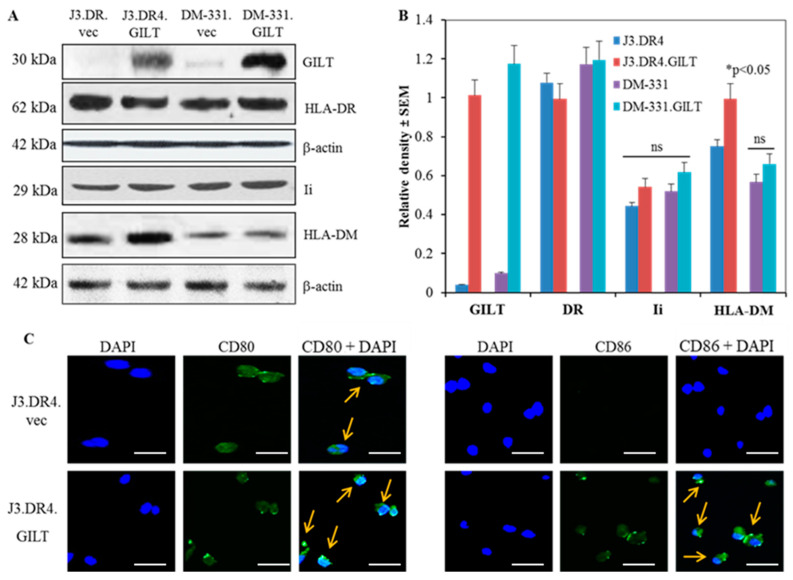
GILT insertion influences the expression of the components of the class II pathway and CD80/CD86 molecules in human melanoma cells. (**A**) Western blot analysis of whole cell lysates from J3.DR4.vec, J3.DR4.GILT, DM-331.vec and DM-331.GILT cells showing protein expression of GILT, HLA-DR, Ii, and HLA-DM. β-actin was utilized as a loading control. (**B**) Densitometric analysis was performed using β-actin as a reference protein band to quantitate relative protein expression. Data are representative of at least three separate experiments. Significant differences were calculated by student’s *t* test; * *p* < 0.05, ns = not significant. (**C**) J3.DR4 cells transfected with empty vector or GILT were simultaneously stained with primary antibodies against CD80 and CD86 proteins, followed by Alexa488-labeled secondary antibody, as described in the methods. Slides were analyzed by Leica TCS SP5 confocal laser scanning microscope using Las-AF software. Representative confocal microscopy images of J3.DR4.vec and J3.DR4.GILT cells indicate increased levels of CD80 and CD86 expression (green) with nuclear staining DAPI (blue), overlapped (arrows) in the right hand corners. Bar = 29.9 μm.

Studies performed in our laboratory and by others have shown that in contrast with many other solid tumors, human melanomas often express detectable levels of HLA class II molecules [50]. Despite their expression of these HLA molecules, melanoma cells often inefficiently process and present Ags to CD4+ T cells, which may contribute to their immune evasion. The reductive cleavage of protein Ags/peptides in acidic endolysosomal compartments is also required before antigenic peptides can be loaded onto class II molecules. Following Ag processing, other important components of the class II pathway such as Ii and HLA-DM can regulate epitope/peptide loading into the HLA class II binding groove [51,52,53]. Ii regulates HLA class II presentation while HLA-DM affects peptide loading onto class II molecules and the presentation of stable class II–peptide complexes to T cells [45]. While melanoma cells that express HLA class II molecules typically express measurable levels of Ii and HLA-DM, the detection of GILT within these tumor lines varies, with some tumors expressing little to no GILT enzyme [15,50]. To examine mechanistically the impact of GILT expression on the HLA class II pathway and Ag presentation in melanomas, two melanoma lines with low to no detectable GILT protein were transduced with a plasmid to promote sustained human GILT expression, or a control empty vector [50]. The parental tumor and GILT-transfected cells included the lines J3.vec, J3.GILT, DM-331.vec, and DM-331.GILT, with GILT expression in these transfected cells confirmed by western blot analysis (Figure 1A). J3.vec and J3.GILT cells were further transduced with HLA-DR4 to generate J3.DR4.vec and J3.DR4.GILT cells. The expression of HLA class II proteins was detected in these cells by western blotting and flow cytometry. The expression of GILT in melanoma cells did not significantly alter intracellular class II protein levels, as determined by western blot analysis (Figure 1A,B). GILT’s effect on Ii and DM protein expression was next assessed by western blot analysis. GILT expression slightly upregulated Ii and DM protein levels in each melanoma cell line (Figure 1A,B), which could slightly impact the loading of antigenic peptides into the class II binding groove. Flow cytometric analysis also confirmed that GILT expression did not influence cell surface expression of class II proteins (MFI: 245.06 vs. 254.16 in J3.DR4.vec and J3.DR4.GILT; MFI: 181.62 vs. 183.60 in DM-331.vec and DM-331.GILT) in melanoma cell lines (Supplementary Figure S1). These data suggest that GILT expression in melanoma cells does not alter surface or intracellular HLA class II protein levels, but that a very marginal increase in Ii or HLA-DM may be detected in tumors expressing high levels of GILT.

### 2.2. GILT Expression Upregulates CD80/CD86 Molecules in Melanoma Cell Lines

A contributing factor to the lack of optimal CD4+ T cell activation is the absence or low levels of the co-stimulatory molecules CD80 and CD86 on melanoma cells [6]. Along with Ag presentation by HLA class II molecules on the surface of melanoma cell lines, the costimulatory signals received by T cells play a key role in enhancing and prolonging the activation of CD4+ T cells. Given the lack of CD40 costimulatory molecule expression by melanomas, the ligation of CD86 or CD80 with T cell CD28 molecules may be even more critical [54,55]. Flow cytometric analysis of these tumors confirmed a slight increase in cell surface expression of CD86 molecules, while J3.DR4.GILT cells also displayed greater CD80 surface expression (Supplementary Figure S2A). Interestingly, GILT expression in melanoma lines resulted in elevated CD86 protein levels detected by western blot analysis (Supplementary Figure S2B), consistent with the immunofluorescence analysis of the J3.DR4.GILT tumor (Figure 1C). Higher CD80 expression was also detected when GILT levels were increased for the J3.DR4.GILT tumor. The increase in CD80/CD86 molecules suggests that GILT impacts the processing of CD80/CD86, and with low GILT they are more rapidly degraded.

### 2.3. GILT Colocalizes with Acidic Cathepsins B and D in Melanoma Cell Lines

**Figure 2:**
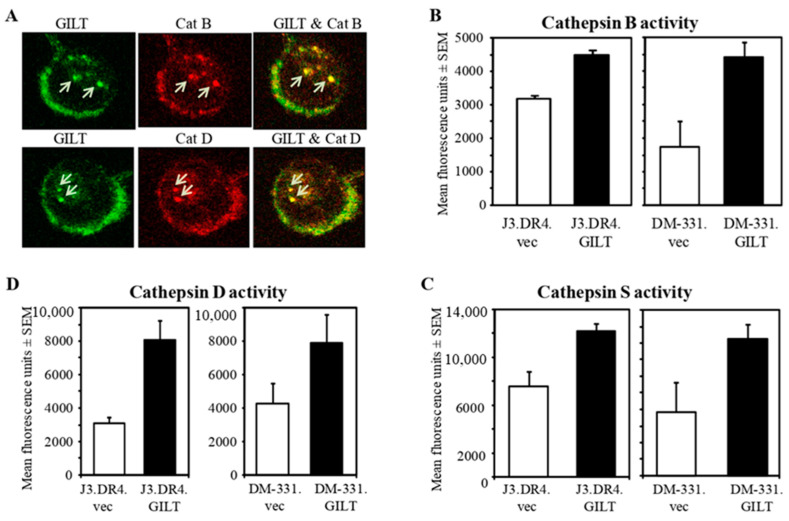
GILT colocalizes with cysteinyl and aspartyl cathepsins and enhances their activities in human melanoma cells. (**A**) J3.DR4.GILT cells were stained with either rabbit anti-CatB or CatD, and co-stained with goat anti-GILT, followed by rhodamine-conjugated anti-rabbit IgG and FITC-conjugated anti-goat IgG antibodies. Live images were acquired using a Leica TCS SP2 AOBS confocal system and processed with Leica software. The colocalization of GILT (green) with CatB (upper panel) and CatD (lower panel) is indicated by the yellow staining and arrows. (**B**–**D**) Cell lysates obtained from J3.DR4.vec, J3.DR4.GILT, DM-331.vec, and DM-331.GILT cells wBar ere analyzed for cathepsins B, S and D activities as described in the methods. Enhanced activities of cysteinyl/asparrtyl cathepsins were detected in melanoma cells expressing GILT, as compared to cells lacking GILT. The experiments were repeated at least three times and results are expressed as mean fluorescence unit ± SEM of triplicate wells.

The importance of acidic cathepsins in class II Ag processing and presentation cannot be overstated. Cysteinyl and aspartyl cathepsins are responsible for the processing of self-Ags into smaller peptides for presentation to CD4+ T cells. We have previously shown that GILT expression upregulates active forms of cathepsins [50,56,57], which may help the processing of Ags/peptides for class II presentation. GILT expression in melanoma cells may thus enhance the reductive cleavage of self-Ags, allowing protein unfolding. These partially processed polypeptides are very susceptible to degradation by acidic cathepsins. Without reductase/cathepsin activity, endogenous and exogenous Ags cannot be efficiently processed into functional epitopes for class II loading, an adaptation tumor cells might exploit to avoid immune detection. To determine if GILT is co-localized with these cathepsins and thus might directly impact their activity, confocal microscopy was employed. Cells were stained with antibodies to detect CatB, CatD, and GILT. In Figure 2A, the overlayed images from J3.DR4.GILT cells indicate that GILT was colocalized with these cathepsins in endolysosomal compartments of melanoma cells, consistent with a potential role for GILT in controlling the endolysosomal redox microenvironment of these proteases.

### 2.4. GILT Expression Enhances the Activity of Cysteinyl Cathepsins B/S and Aspartyl Cathepsin D in Melanoma Cell Lines

Due to the colocalization of GILT with acidic cathepsins, the activity of cysteinyl cathepsin Cat B was analyzed in J3.DR4.vec, J3.DR4.GILT, DM-331.vec and DM-331.GILT cells. Interestingly, Cat B activity was significantly increased (J3.DR4.vec = 3873 vs. J3.DR4.GILT = 4499.3, *p* ≤ 0.05; DM-331.vec = 622.67 vs. DM-331.GILT = 672, *p* ≤ 0.01) in these two different melanoma cell lines upon transfection with GILT (Figure 2B). Similarly, Cat S activity was significantly increased in GILT-transfected J3.DR4.GILT (12,348 vs./7730, *p* ≤ 0.01) and DM-331.GILT (7477 vs. 6521, *p* ≤ 0.05) cells (Figure 2C). However, there were no significant changes in Cat L activity in DM-331.vec and DM-331.GILT cells, and Cat L activity was lower in J3.DR4.GILT cells when compared with J3.DR4 cells (data not shown). Both Cat S and Cat L can process Ii to facilitate HLA class II maturation and the acquisition of antigenic peptides [50]. Collectively, these results suggest that GILT expression enhances the activity of cysteinyl cathepsins B and S in human melanoma cells. This increase in protease activity in GILT-expressing melanomas may alter the pool of functional epitopes available for HLA class II molecules, contributing to enhanced Ag presentation.

Cathepsin D is an integral acidic protease involved in Ag processing, and exhibits site-specific cleavage and splicing of Ags, which is important for short peptide loading onto HLA class II molecules [48,49]. An increase in cathepsin D expression and activity would aid melanoma cells in enhanced Ag processing and the presentation of class II–peptide complexes to CD4+ T cells. GILT expression upregulated cathepsin D enzymatic activity in two distinct melanoma cell lines (Figure 2D), pointing again to a role for GILT in altering the spectrum of functional peptides for class II presentation and CD4+ T cell recognition.

### 2.5. Mass Spectroscopic Identification of Peptides with Modified Cysteine Residues in Melanoma Cell Lines Dependent on GILT Expression

**Figure 3:**
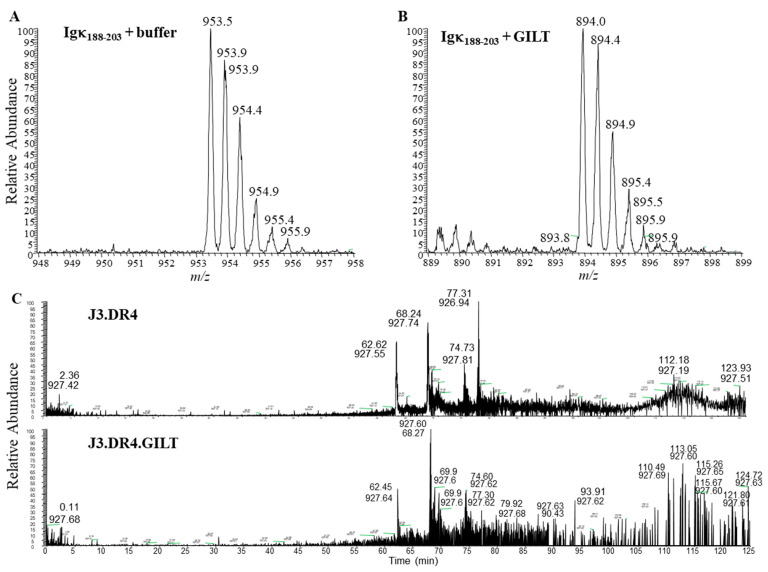
Mass spectroscopy and fragmentation of cysteinylated versus reduced Igκ_188–203_ peptides. (**A**) Cysteinylation of the κ_188–203_ peptide was determined by the detection of ionized species at 954 *m/z*. (**B**) GILT catalyzed the reduction of cysteinylated-κ_188–203_ as ionized species at 894 detected. (**C**) Extracted ion chromatograph of peptides generated by J3.DR4 and J3.DR4.GILT cells. LC-MS mass spectrometry was performed on supernatant obtained from J3.DR4 and J3.DR4.GILT cells fed with IgG. The data suggest that J3.DR4.GILT cells generated a greater pool of reduced peptides.

**Figure 4:**
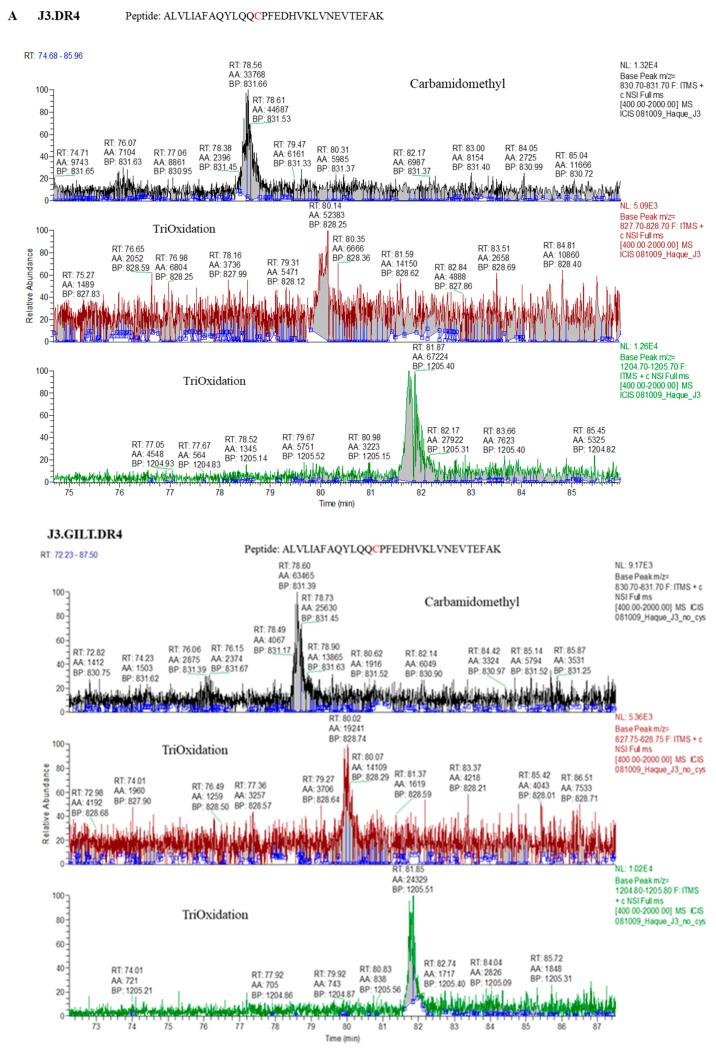

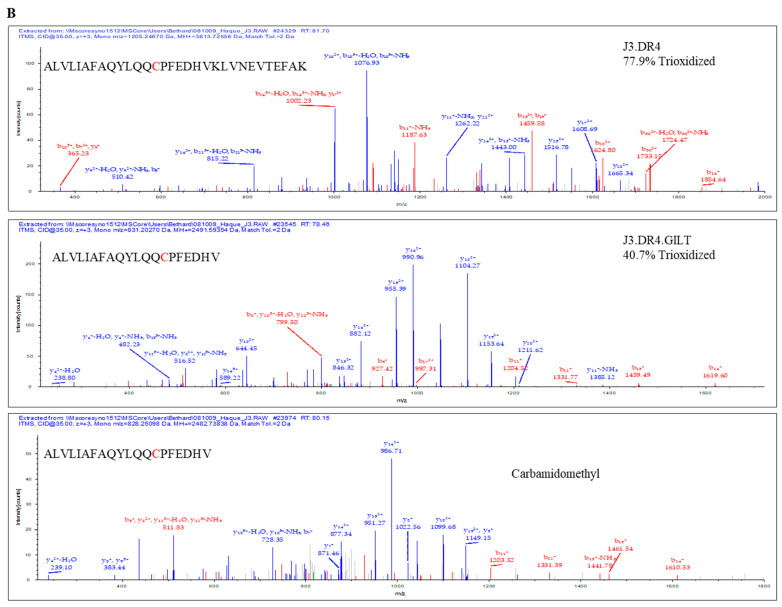
Percent occupation of cysteine oxidation in supernatants obtained from cells with or without GILT. (**A**) J3.DR4 and J3.DR4.GILT cells were fed with IgG in serum-free HBSS for 4 h. Supernatants were collected by centrifugation and analyzed by mass spectroscopy as described in the methods. Extracted ion chromatograph (XIC) of the peptide ALVLIAFAQYLQQCPFEDHVK identified in supernatants obtained from cells±GILT; 831.66 is the mass of the +3 charge state for the peptide ALVLIAFAQYLQQCPFEDHVK with the cysteine chemically modified by iodoacetamide (+57); 828.25 is the +3 charge state of ALVLIAFAQYLQQCPFEDHVK, which contains a tri-oxidized cysteine; 1205.4 is +3 charge state of the peptide ALVLIAFAQYLQQCPFEDHVKLVNEVTEFAK, which contains a tri-oxidized cysteine and 1 trypsin missed cleavage. The percent occupation of oxidation was calculated using the area under the curve from the XIC (the total area of the oxidized peptides divided by the total area of the peptide ×100). Cells expressing GILT had a 40.7% tri-oxidized cysteine, while cells lacking GILT had a significantly higher level of oxidation with 77.9% of the peptide being tri-oxidized. (**B**) Tandem MS spectra (MS/MS) of the reduced and oxidized forms of the peptides ALVLIAFAQYLQQCPFEDHVK and ALVLIAFAQYLQQCPFEDHVKLVNEVTEFAK, with the cysteine detected as either carbamidomethylated or tri-oxidized. The top spectra identified ALVLIAFAQYLQQCPFEDHVKLVNEVTEFAK with the cysteine tri-oxidized with an Xcorr value of 5.39. The site of oxidation was confirmed by the presence of the b14 ions 1610.8 (+1) and 805.9 (+2) and the y18 ion 805.9 (+2). The middle tandem MS spectrum was identified as ALVLIAFAQYLQQCPFEDHVK with a trioxidized cysteine and an Xcorr value of 3.36. The site of oxidation was confirmed by the presence of the b14 ions 1610.8 (+1) and 805.9 (+2) and the y8 ions 1022 (+1) and 511.7 (+2). The bottom MS/MS spectra was identified with an Xcorr of 4.78 as the peptide ALVLIAFAQYLQQCPFEDHVK, with the cysteine carbamidomethylated. The modification detected by mass spectral analysis was localized by the presence of b14 ions 1619 (+1) and 810 (+2), and the y8 ion 516(+2).

Because GILT expression enhances cathepsins’ activity, we next sought to examine whether GILT-positive melanoma cell lines generate a distinct pool of functional peptides as compared to GILT-negative tumor cells. Melanoma cell lines J3.DR4.vec and J3.DR4.GILT were incubated with the whole Ag IgG for 6 h. The spent cell media was collected for detecting processed and released IgG-derived epitoeps. The levels of the reduced IgGκ_188–203_ peptide were more abundant in the media of J3.DR4.GILT tumor cells as compared to J3.DR4.vec cells, confirming GILT’s ability to functionally reduce this antigenic epitope following its processing (Figure 3A,B). Mass spectroscopic analysis also suggested that the J3.DR4.GILT cells produced a greater array of diverse peptides as compared to J3.DR4 cells (Figure 3C). Further analyses by LC-MS showed that a greater pool of oxidized peptides (77.9%) was generated by tumor cells lacking GILT, by comparing the structure of a cysteine-containing peptide released by each cell (Figure 4A,B). By contrast, only 40.7% of the oxidized peptide was detected in melanoma cell lines expressing GILT. The peptide ALVLIAFAQYLQQCPFEDHVK with the cysteine was chemically modified by iodoacetamide. Interestingly, cells expressing GILT had less oxidized cysteines, while cells lacking GILT showed a significantly higher level of oxidation of the peptide being tri-oxidized. The data also suggest that no major alterations were observed when a non-cysteine-containing peptide was analyzed. These data suggest that GILT expression in melanoma cells facilitates the generation of a greater pool of reduced peptides, some of which may bind to HLA class II molecules and activate CD4+ T cells. 

### 2.6. GILT Expression in Melanoma Cell Lines More Broadly Impacts Ag Processing

**Figure 5:**
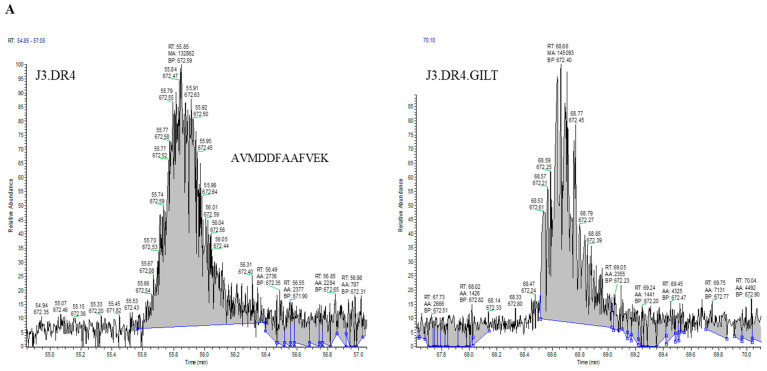

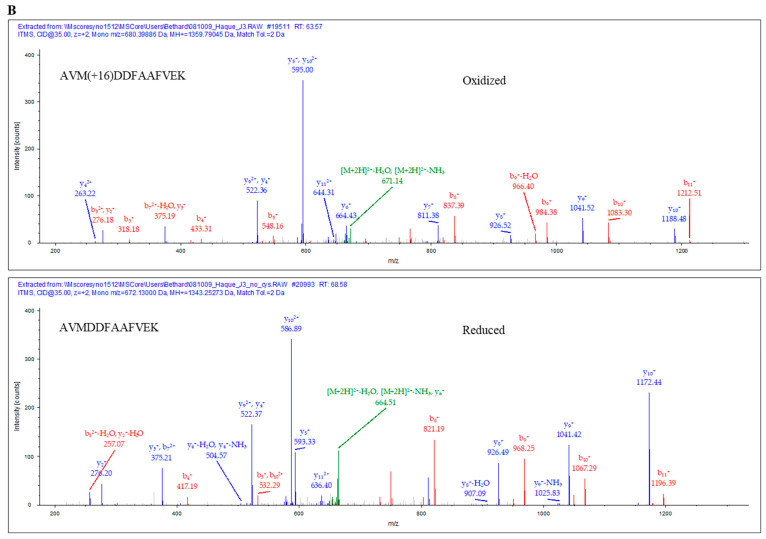
Percent occupation of methionine oxidation in supernatants obtained from cells with or without GILT. Cells (J3.DR4 and J3.DR4.GILT) were fed with IgG in serum-free HBSS for 4 h. Supernatants obtained were analyzed by mass spectroscopy. (**A**) Extracted ion chromatograph (XIC) of the peptide AVMDDFAAFVEK identified in cell supernatants obtained from J3.DR4 and J3.DR4.GILT cell; 672.5 was identified as the reduced form of peptide AVMDDFAAFVEK, and determined to be the +2 charge state. The oxidized form of this peptide, 680.5 (+2 charge state), was below the quantitation limit as a peak was unable to be extracted to calculate the area. Based on the areas of the unoxidized form of this peptide, there is no significant change in oxidation of methionines in J3.DR4.GILT supernatant. (**B**) Tandem MS spectra (MS/MS) of the reduced (672.1) and oxidized (680.1) forms of the peptide AVMDDFAAFVEK. The top spectra represent the oxidized form of the peptide AVMDDFAAFVEK. This peptide was identified with an Xcorc value of 3.15. Localization of the oxidized amino acid was determined by the presence of the b3 ion at 318.3 and the y10 ions at 1188.5 (+1) and 594.7 (+2). The bottom spectrum was identified as the reduced peptide AVMDDFAAFVEK with an Xcorr of 3.53.

Since GILT expression enhanced both aspartyl and cysteinyl cathepsin activity in melanoma cell lines, it was pertinent to investigate whether GILT expression in melanoma cells could generate more functional peptides for HLA class II presentation and T cell recognition. J3.DR4 ± GILT cells were incubated overnight with the whole IgGκ. Spent cell media was collected, filtered with a 0.45 µm filter, and analyzed by mass spectrometry. The data obtained showed an increase in the generation of the peptide AVMDDFAAFVEK in GILT-expressing melanoma cells (~2× more peptide in J3.DR4.GILT cells, based on Peak area analyzed). While the top spectra represent the oxidized form of the peptide AVMDDFAAFVEK, the bottom spectrum was identified as the reduced peptide AVMDDFAAFVEK. This is a significant finding, as analyzed by mass spectroscopy. In tumor cell lines with GILT expression, only the reduced forms of this peptide (AVMDDFAAFVEK) were detected without methionine modification, suggesting that overall processing of the Ag was altered (Figure 5A,B). These data again suggest that tumor GILT expression impacts Ag processing.

### 2.7. GILT Expression Enhances HLA Class II-Mediated Antigen Processing and Presentation

**Figure 6:**
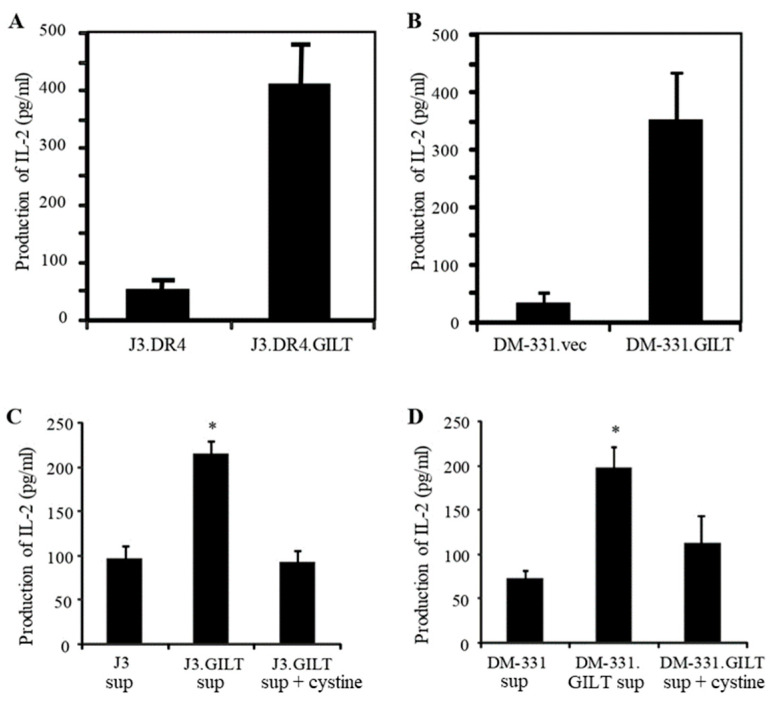
GILT expression enhances HLA class II-mediated antigen presentation and CD4+ T cell recognition of tumors by producing a greater pool of functional epitopes. (**A**,**B**) Human melanoma cell lines J3.DR4.vec, J3.DR4.GILT, DM-311.vec, and DM-311.GILT were incubated with whole Igκ Ag overnight at 37 °C. Cells were then washed, and co-cultured with the κ_188–203_ peptide-specific CD4+ T cell hybridoma line (2.18a) for 24 h. The T cell production of IL-2 in the culture supernatant was measured by ELISA. The production of IL-2 is used as an indication of Ag presentation and T cell recognition. Data are expressed as the mean ± SD, shown in pg/mL of triplicate wells of at least three independent experiments. * *p* < 0.01. (**C**,**D**) Melanoma cell lines J3.DR4.vec, J3.DR4.GILT, DM-331.vec, and DM-331.GILT were incubated overnight with whole Igκ Ag in HBSS at 37 °C. Cell supernatants were transferred to paraformaldehyde fixed J3.DR4.vec (**C**) and DM-331.vec (**D**) cells overnight at 37 °C. Supernatants from GILT-expressing cells were also incubated with L-cystine (290 μM) as described. Cells were then washed and co-cultured with the κ_188–203_ peptide-specific CD4+ T cell hybridoma 2.18a for 24 h. The T cell production of IL-2 was quantitated by ELISA. Data are representative of at least three separate experiments. * *p* < 0.001.

We have previously reported that GILT expression could significantly improve CD4+ T cell recognition of human melanoma cells [15]. In an effort to further understand the role of GILT in the immune recognition of human melanomas, several melanoma lines were incubated with the native Ag Igκ. After incubation, melanoma cell lines were washed and cocultured with the T cell hybridoma line 2.18a, which recognizes the Igκ_188–203_ epitope. Analysis of functional Ag presentation by the melanoma cell lines revealed that GILT expression enhanced CD4+ T cell recognition of antigenic epitopes displayed in the context of HLA class II proteins on tumor cells (Figure 6A,B). In parallel, cell surface HLA-DR4 molecules were analyzed by flow cytometry, which showed that GILT expression did not alter cell surface HLA-DR4 expression in melanoma cell lines (Supplementary Figure S3A,B). Co-cultures of melanoma cell lines (e.g., DM-331) and Th1 cells (2.18a cells) did not influence GILT expression in DM-331 cells (Supplementary Figure S4). In a prior study, increased oxidation of cysteine residues in the antigenic peptide was found to impair T cell recognition [15]. Our current study suggests that the expression of GILT can control Ag processing, as well as the post-translational modification of antigenic peptides. To further examine GILT’s role in Ag processing, melanoma cell lines were incubated with the whole Ag Igκ, followed by the collection of the spent media by centrifugation. The spent media from each cell line was added to aldehyde-fixed J3.DR4.vec or DM-331.vec cells to facilitate the capture of newly formed Ig epitopes by class II molecules on each of these fixed cells. Aldehyde fixation prevents Ag internalization and processing, so that only peptides formed in the spent media could be captured by class II molecules and presented to T cells specific for a cysteine-containing epitope within the Ag Igκ. J3.DR4.vec cells incubated with spent media obtained from IgG-fed J3.DR4.vec cells minimally stimulated T cells (Figure 6C). By contrast, J3.DR4.vec cells incubated with spent media obtained from IgG-fed J3.DR4.GILT cells were more effective in activating CD4+ T cells. The addition of oxidized cystine to the spent media of the J3.DR4.GILT cells promoted the oxidation of the Igκ epitope and reduced T cells recognition (Figure 6C). Strikingly, similar results were observed with the DM-331 tumor cell line (Figure 6D). Fixed (paraformaldehyde, 1%) or unfixed J3.DR4 and DM-331 cells with or without GILT were also incubated with Igκ (145–159) peptide, followed by coculturing with the peptide-specific hybridoma line 1.21 (Supplementary Figure S5). The T cell production of IL-2 was quantitated by ELISA. These data show that the fixation of melanoma cells did not significantly alter peptide presentation to T cells. Together, these results suggest that tumor cell GILT expression significantly impacts Ag processing and CD4+ T cell recognition, and may contribute to the future designing of cancer vaccines.

**Figure 7:**
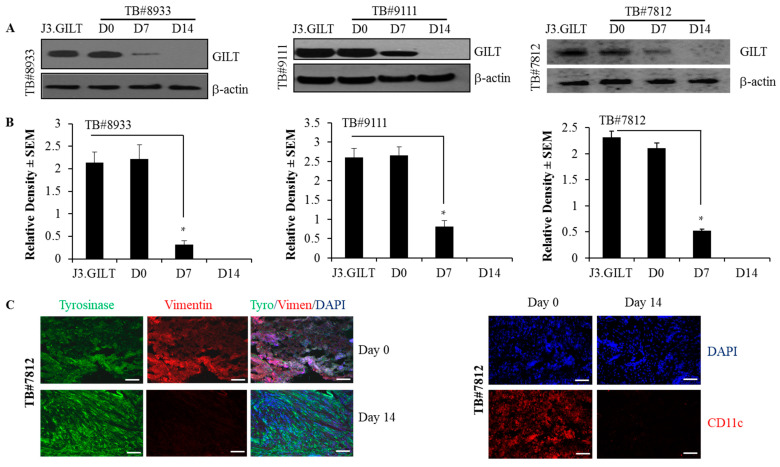
GILT expression is detected in tumor-infiltrating cells and is lost in human metastatic melanoma. Human melanoma patient samples TB#8933, TB#9111, and TB#7812 were minced into cubes (approx. 1 mm) and placed into T75 flasks with complete DMEM containing 10% FBS and 1 mg/mL collagenase. These tumor tissues were incubated at 37 °C for 24 h, washed, suspended in complete DMEM with no collagenase in the medium, and cultured for at least 14 days as described in the methods. (**A**) Samples from day 0, day 7 and day 14 were subjected to western blot analysis. Western blot analysis showing differential GILT expression at day 0 (high) and day 7 (low), and the expression was lost at day 14 in metastatic tumors. J3 cells transfected with GILT (J3.GILT) were used as a positive control. (**B**) Densitometric analyses were performed using β-actin as a reference protein band to quantitate relative GILT protein expression in human metastatic melanoma at different time points. Data are representative of at least three separate experiments. * *p* < 0.001. (**C**) Immunohistochemical analyses of TB#7812 cultures for tyrosinase (melanoma), vimentin (fibroblast) and CD11c (dendritic cells) markers at day 0 and day 14. Bar = 100 μM.

The analysis of human melanoma patient samples showed that high levels of GILT proteins were detected in melanoma tissues at day 0. However, after the culturing of tumor tissues in media for 7 days, GILT expression went down significantly, and at day 14, GILT expression was almost undetectable (Figure 7A,B). Melanoma cultures were also stained for fibroblast (vimentin), melanoma (tyrosinase), and dendridic cell (CD11c) markers at day 0 and day 14 by immunohistochemistry (Figure 7C). The data show that the melanoma tissue culture was positive for vimentin, tyrosinase, and CD11c at day 0. However, at day 14, the culture was only positive for tyrosinase, and it was negative for vimentin and CD11c. These data strongly suggest that the population of melanoma is devoid of APC (e.g., dendritic cells) and fibroblasts, as these infiltrating cells were undetected by immunohistochemistry at day 14. These data also indicate that infiltrating cells in melanoma tumor tissues may express GILT, and malignant melanoma cells may lack GILT or express very low levels of this enzyme in the tumor, which could be an immune escape mechanism for metastatic melanoma.

## 3. Discussion

An important characteristic in the immune evasion of melanoma tumors is the alteration of tumor Ag presentation, or interruption of the HLA class II Ag presentation pathway. Restoring the HLA Class II presentation pathway in melanoma cells has been shown to support tumor destruction [15,50]. However, melanoma cells lack an important enzyme, GILT, perturbing epitope presentation via the HLA Class II machinery. Therefore, investigation into the role of GILT in epitope generation for the class II pathway and its involvement in stimulating CD4+ T cell recognition is essential in the development of improved immunotherapeutic strategies. In this study, we have shown that GILT enhances multiple components of the HLA class II pathway, such as Ii and HLA-DM. The expression of Ii is important for stabilizing HLA class II molecules by effectively forming a trimer within the endoplasmic reticulum, thus regulating HLA class II presentation [58,59,60]. HLA-DM acts as a peptide editor upon the class II DR complex to determine if the correct epitope binds to the binding groove [61,62]. Thus, DM can accelerate the reaction from unstable to stable class II–peptide complex for immune recognition [50]. GILT expression also enhanced the intracellular expression of CD80/CD86, but this was not translated on the cell surface. An increase in intracellular CD80/CD86 does correlate with the expression on the cell surface [63]. However, this upregulation of costimulatory molecules by GILT could lead to a reduction in T cell tolerance [64] and immune avoidance by melanoma cells. 

Professional APCs express high levels of GILT, which is crucial for Ag processing and presentation to CD4+ T cells. However, melanoma and other tumors express low to no detectable levels of GILT, which could be correlated to the tumor cells’ inability to display a broad repertoire of functional class II–peptide complexes on their cell surface to T cells [65]. Professional APCs possess all the machinery to optimally process and present antigens to T cells, while malignant tumor cells lack some of the processing machinery needed to generate the large repertoire of functional HLA peptide complexes necessary for a robust CD4+ T cell response [15,50,66]. By further understanding the steps and factors involved in Ag processing reactions, new potential targets for immunotherapy may emerge. This study suggests that GILT expression in melanoma cells enhances HLA class II antigen processing. GILT expression also enhances the enzymatic activity of cathepsins B, S, and D, which correlates with our previous finding that GILT causes an increased expression of active forms of cysteinyl and aspartyl cathepsins [50]. Intracellular proteases, such as cysteinyl cathepsins B and S, are essential for the degradation of endogenous and exogenous Ags, and each serves an important role in the processing of peptides for presentation via HLA class II molecules. Aspartyl cathepsin D activity was significantly upregulated, which may contribute to the enhanced processing of self-Ags/peptides. This suggests that Ags, both endogenous and exogenous, may be readily processed by GILT-expressing tumor cells, which could allow for more functional epitopes being available for presentation via the class II pathway [67]. The final event would then end with an increase in immune recognition and a stronger anti-tumor immune response. Since melanoma express their own tumor Ags, such as NY-ES0-1, gp100, and tyrosinase [68,69,70], the increase in enzymatic activity by GILT-expressing melanoma cells could be exploited for the induction of tumor Ag-specific T cells. A further understanding of GILT’s role in antigen processing and presentation in melanoma may lead to the development of new and more effective immunotherapeutics against metastatic melanoma.

Studies have shown that melanoma cells express undetectable to low levels of GILT, thus leading to immune evasion [15]. Nguyen et al. recently presented data suggesting that GILT is upregulated in human melanoma [34]. In that study, human primary and metastatic melanomas and nevi were examined via immunohistochemical analysis, and the results showed that MHC class II and GILT expressions in melanocytes were increased in primary and metastatic melanomas compared with nevi. However, we investigated GILT expression in human melanoma tumors and found GILT protein significantly decreased in culture over time. Allowing tumor cells to culture and persist while tumor-infiltrating APCs are removed implies that GILT protein is expressed in infiltrating macrophages and dendritic cells, while it is lost in the primary tumor. We have verified that GILT expression is lost in human melanoma tumors, and have shown a need to investigate the role of GILT in the recognition of malignant melanoma.

Functional antigen presentation is at the root of generating optimum T cell responses. The expression of GILT can play an important role in processing modified or oxidized Ags/peptides for the enhanced CD4+ T cell recognition of tumors. Unfortunately, the majority of melanomas either lack GILT or express this protein at a reduced level, which may help the immune evasion of melanoma. Although other studies reported GILT expression in human melanoma tumors by immunohistochemistry [34], our study suggests that infiltrating cells, but not metastatic melanoma, express GILT, and that GILT-expressing melanomas are a better target for CD4+ T cells. The presence of GILT influences several key components of the class II pathway in melanoma. It enhances the expression of the cathepsins and increases Ag processing and presentation, leading to the increased stimulation of CD4+ T cells. Other studies have shown that GILT is important in the development of the overall T cell response to protein antigens that contain disulfide bonds [15,30,50].

With the combination of GILT-facilitated reduction with proteolysis in the endosomal/lysosomal compartments in melanoma cells, it is safe to assume that the generation of class II epitopes would be enhanced, but this had never been tested. We present evidence that the pool of antigenic peptides/epitopes generated by the presence of GILT has increased functionality, illustrating GILT’s ability to reduce oxidized or cysteinylated peptides to a more functional form. This is also supported by the observed increase in GILT/cathepsins. We have previously shown that a large number of melanoma cells do not express GILT, and this may result in immune evasion of melanoma via the MHC class II pathway. However, it is possible that a particular group of melanomas may express GILT and result in immune recognition and increased patient survival. Further studies are needed to determine whether a particular group of melanoma patients, or populations within the same tumor, differentially express GILT and influence the immune detection of tumors. The presence of GILT produced more functional epitopes from whole Igκ, which then was able to increase CD4+ T cell recognition and IL-2 production. This action was inhibited when the supernatant was incubated with the presence of cystine, which suggests that cysteinylation inhibits peptide presentation, and GILT expression may overcome the inefficient processing of self-Ags/peptides. 

## 4. Materials and Methods

### 4.1. Cell Lines and Tumors

The human melanoma cell line DM-331 was maintained in complete RPMI-1640 (Invitrogen, Grand Island, NY, USA) medium supplemented with 10% fetal bovine serum (FBS) (Hyclone, Logan, UT, USA), 50U/mL penicillin, 50 μg/mL streptomycin (Mediatech Inc., Manassas, VA, USA), and 1% L-glutamine (Mediatech, Hsinchu, Taiwan) [71]. The human melanoma cell line J3 was maintained in complete IMDM with 10% heat-inactivated bovine growth serum (BGS) (Hyclone), 50 U/mL penicillin, 50 μg/mL streptomycin (Mediatech), and 1% L-glutamine (Mediatech) [15]. The T cell hybridoma cell lines 2.18a (specific for κ_188–203_ peptide) and 1.21 (specific for κ_145–159_ peptide) were cultured in complete RPMI 1640 with 10% FBS, 50 U/mL penicillin, 50 μg/mL streptomycin, 1% L-glutamine and 50 μM β-mercaptoethanol (β-ME) [15,72]. DM-331 cells were also co-cultured with Th1 cells (2.18a) and subjected to western blotting for GILT protein.

For human melanoma patient samples, institutional approval was first obtained, and three patient samples (TB#8933, TB#9111, and TB#7812) were received from our Hollings Cancer Center at the Medical University of South Carolina (MUSC). TB#8933 is an invasive malignant melanoma (staging T1b, N3), TB#9111 is a metastatic melanoma (staging T0, N1), and TB#7812 is also a metastatic melanoma (staging T4b, N3) as analyzed by the Biorepository & Tissue Analysis Shared Resource of the Hollings Cancer Center at MUSC. While all three melanoma tumors are metastatic, TB#8933 is more invasive. Melanoma tumor tissues were minced into cubes (approx. 1 mm) and placed into T75 flasks with complete DMEM (Dullbecco’s modified Eagle’s medium, invitrogen) containing 10% FBS, and 1 mg/mL collagenase (Sigma, St. Louis, MO, USA). These tumor tissues were incubated at 37 °C for 24 h, washed, and suspended in complete DMEM with no collagenase in the medium. Culture medium was changed every two days, and the cells were sub-cultured into different flasks and harvested at day 0, day 7 and day 14; they were then analyzed by western blot analysis for GILT protein. 

### 4.2. Cell Transduction and Transfection

The human melanoma J3 cell line was transduced using retroviral vectors for constitutive expression of HLA-DR4 (DRB1*0401) with linked drug selection markers for hygromycin and histidinol resistance [15]. The expression of surface HLA-DR4 complexes on cells was confirmed by flow cytometric analysis using the DR4-specific mAb, 359-F10 [73]. The J3.DR4 cell line was further transfected with an empty vector or GILT cDNA to generate J3.DR4.GILT [15,71]. The human melanoma cell line DM-331, which constitutively expresses HLA-DR4 molecules on the cell surface, was also transfected with empty vectors or GILT as described [71]. The expression of GILT was confirmed by western blot analysis.

### 4.3. Antigens, Peptides, and Antibodies

The whole Igκ was purchased from Sigma-Aldrich. The human IgGκ (188–203) peptide (sequence KHKVYACEVTHQGLSS) and IgGκ (145–159) peptide (sequence: KVQWKVDNALQSGNS) were produced by Fmoc technology and an Applied Biosystems Synthesizer [30,72]. Peptide purity (>99%) and sequence were analyzed by reverse phase HPLC purification and mass spectroscopy. The primary antibodies used were human GILT, HLA-DM, CD86, Cat B, Cat D, Cat S, Cat L and β-actin (Santa Cruz Biotechnology Inc., Santa Cruz, CA, USA), tyrosinase (ab180753, Abcam), vimentin (V9, ThermoFisher Scientific, Waltham, MA, USA), and CD11c (N418, ThermoFisher Scientific). HLA-DR (L243) and Ii (Pin 1.1) were obtained from Janice Blum (Indiana University School of Medicine, Indianapolis, IN, USA). The secondary antibodies used were horseradish peroxidase conjugated anti-mouse (Pierce, Rockford, IL, USA), anti-rabbit or anti-goat IgG (Santa Cruz). 

### 4.4. Western Blot Analysis

J3.DR4.vec, J3.DR4.GILT, DM-331.vec, and DM-331.GILT lines were cultured in complete RPMI, washed, and cell lysates were obtained using a standard lysis buffer (10 mM Trizma base, 150 mm NaCl, 1% Triton-X 100) [15,35,71]. Human melanoma patient samples TB#8933 and TB#9111 were also subjected to western blotting for GILT protein expression (Santa Cruz antibody). Equal protein concentrations from designated cell lysates were separated on a 4–12% Bis/Tris NuPage gel (Invitrogen, Grand Island, NY, USA). Proteins were transferred onto a nitrocellulose membrane (Pierce) and probed with antibodies for the expression of GILT, HLA-DR, Ii, HLA-DM, CD80, and CD86. The secondary antibodies used were horseradish peroxidase conjugated anti-mouse (Pierce), anti-rabbit, or anti-goat IgG (Santa Cruz). A monoclonal antibody for β-actin (Santa Cruz) was used as a protein loading control. Relative protein expression was also assessed using a Bio-Rad scanning densitometer, and further stated as a ratio of specific proteins expressed/β-actin for each sample [50,56,57].

### 4.5. Flow Cytometry

J3.DR4.vec, J3.DR4.GILT, DM-331.vec, and DM-331.GILT cells were cultured and washed with staining buffer (PBS + 1% heat-inactivated BGS) (Hyclone) and resuspended in a binding buffer (cat. No. 556454, BD Bioscience: 0.1 M HEPES (pH 7.4), 1.4 M NaCl_2_, and 25 mM CaCl_2_). Cells were stained with anti-DR, anti-CD80, and anti-CD86 (Santa Cruz), followed by fluorescence labeled secondary antibodies. Samples were then analyzed on a FACScan using CellQuest software (BD Bioscience, Mountain View, CA, USA) for cell surface expression of class II, CD80/CD86 molecules with NN4 and other appropriate isotype controls [50,56,57].

### 4.6. Confocal Microscopy

J3.DR4.vec, J3.DR4.GILT cells and melanoma tissues were cultured on glass coverslips (cat# 12-545-80, Fisher Scientific Co., Pittsburgh, PA, USA). Cells and melanoma cultures were washed and fixed with (1:1) acetone/methanol mixture for 10 min at room temperature. Cells were then permeabilized with 0.1% Triton-X 100 for 15 min, blocked with 5% normal serum for 10 min, and incubated with the CD80 and CD86 antibodies at 37 °C for 1 h. Following incubation, cells were washed twice with 1% BSA (cat. No. 2930, OmniPur, EMD, Cincinnati, OH, USA) in PBS, and incubated with Alexa 488-FITC conjugated donkey anti-goat Ig (Santa Cruz) and Alexa 543-rhodamine conjugated goat anti-mouse Ig (Santa Cruz) for 1 h. For the staining of TB#7812 melanoma cultures, primary antibodies including tyrosinase (ab180753, Abcam), vimentin (V9, ThermoFisher Scientific), and CD11c (N418, ThermoFisher Scientific) were used followed by incubation with appropriately matched secondary antibodies. Cells were also counter-stained with DAPI (4′-6-Diamidino-2-phenylindole) for nuclear localization. The slides were mounted in fluorescent mounting medium G (South Biotechnology, Inc., San Francisco, CA, USA), observed with a 63× N.A.1.4 oil immersion objective lens, and analyzed by a Leica TCS SP5 confocal laser scanning microscope using Las-AF software (Leica Lasertechnik, Wetzlar, Germany) [50].

### 4.7. Cathepsin Bioassay

Cathepsin B, S, L, and D activity assay kits were purchased from BioVision, Inc. (Biovision, Milpitas, CA, USA). Cathepsin B, S, L, and D activity was measured in melanoma cell lines J3.DR4.vec, J3.DR4.GILT, DM-331.vec, and DM-331.GILT according to the manufacture’s specified protocol (BioVision). Experiments were performed in triplicates and repeated at least three times. Results and standard deviations for a single representative experiment are shown.

### 4.8. Liquid Chromatography-Mass Spectroscopy (LC-MS)

J3.DR4.vec and J3.DR4.GILT cells were fed with IgG κ for 24 h, cell media was collected, and the IgG κI peptide (sequence KHKVYACEVTHQGLSS) was analyzed by capillary liquid chromatography using an Applied Biosystems 140D solvent delivery system. Samples were applied directly to 300 μM diameter fused silica capillaries packed with Vydac C18 resin and separated with gradients of buffer A (2% acetonitrile and 98% H_2_O containing 0.2% isopropanol, 0.1% acetic acid and 0.001% trifluoroacetic acid) and buffer B (95% acetonitrile and 5% H_2_O containing 0.2% isopropanol, 0.1% acetic acid and 0.001% trifluoroacetic acid). Peptide was eluted at a flow rate of 7 μL/miN directly into the electrospray ionization source of a Finnigan LCQ mass spectrometer. Nitrogen was used as the sheath gas with a pressure of 35 psi with no auxiliary gas. Electrospray ionization was conducted with a spray voltage of 4.8 kV, a capillary voltage of 26 V, and a capillary temperature of 200 °C. Spectra were scanned over an *m/z* range of 200–2000. Base peak ions were trapped using the quadruple ion trap and further analyzed with a high resolution scan (zoom-scan) using an isolation width of 3 *m/z* and collision-induced dissociation scans with a collision energy of 40.0. 

### 4.9. Sample Preparation for LC MS/MS

Cell elusions were reduced with DTT and alkylated with 55 mM iodoacetamide. The protein was digested with 100 ng of trypsin (Sigma, proteomics grade) overnight at 37 °C. The digested sample was then desalted using a C18 ziptip (Millipore, Burlington, MA, USA) following the manufacturer’s protocol. The eluent was dried, and peptides were re-suspended in 5 µL of mobile phase A (98% water, 2% acetonitrile, 0.1% formic acid) and transferred to an auto sampler vial for LC-MS/MS analysis.

### 4.10. LC MS/MS Analysis-LTQ XL

Trypsin-digested samples were analyzed via liquid chromatography (LC)–electrospray ionization (ESI)–tandem mass spectrometry (MS/MS) on a linear ion trap mass spectrometer (LTQ, Thermo) coupled to a Dionex U3000 nano LC system. A 25 cm × 75 μm C-18 reversed phase LC column (packed in house, with Waters ODS C18, 5u) was utilized with a 120 min gradient from 2% acetonitrile, 0.2% formic acid to 50% acetonitrile, 0.2% formic acid. Data-Dependent Analysis was utilized on the LTQ to perform MS/MS on the 10 most intense ions in each MS spectra with a minimum ion count of 1000. 

### 4.11. HLA Class II Antigen Presentation Assays

J3.DR4.vec, J3.DR4.GILT, DM-331.vec, and DM-331.GILT cells (5 × 10^5^ cells/mL) were cultured at 37 °C in culture media in a 96-well plate (cat# 3595, Corning Incorporated). Whole Igκ Ag (10 μM) was added to the appropriate wells and incubated overnight. Cells were then washed and cocultured with the κ_188–203_ peptide-specific T cell hybridoma (2.18a) for 24 h. Following incubation, the plates were stored at −80 °C until testing for IL-2 production. Cells +/−GILT were also incubated with whole IgGκ overnight at 37 °C. Supernatants were collected by centrifugation, filtered with a 0.45 μM filter and incubated with J3.DR4.vec and DM-331.vec cells overnight. Supernatants obtained from GILT-expressing cells were also incubated in the presence of oxidized cysteine (290 μM). Cells were then fixed with 1% paraformaldehyde, washed and cocultured with the κ_188–203_ peptide-specific T cell hybridoma (2.18a) for 24 h. J3.DR4.vec, J3.DR4.GILT, DM-331.vec, and DM-331.GILT cells (5 × 10^5^ cells/mL) were also incubated with κ_145–159_ peptide, fixed or unfixed, washed and cocultured with the κ_145–159_ peptide-specific T cell hybridoma (1.21) for 24 h. T cell production of IL-2 was measured by enzyme-linked immunosorbent assay (ELISA) according to the manufacturer’s instructions (R&D Systems, Minneapolis, MN, USA) [15]. Anti-IL-2 was purchased from Sigma-Aldrich. All assays were repeated at least three times.

### 4.12. Database Searching and Quantitation

The raw data were searched with Proteome Discoverer 1.4. The Human IPI v3.72 database was used. Static modification of carbomidomethyl on cysteines and variable modifications of methionine oxidation and the oxidation, di-oxidation, tri-oxidation, and cysteinylation of cysteines were included. Protein identifications must have 2 unique peptides with an Xcorr vs charge state >1.5, 2.0, 2.5 for +1, +2, and +3 ions, and a good match for at least 4 consecutive y or b ion series from the MS/MS spectra to be considered a positive identification.

Peptides identified from the above analysis that were detected in both the oxidized and reduced states were analyzed further for changes in oxidation with treatment by GILT. Xcalibur (Thermo, Waltham, MA, USA) was used to display the extracted ion chromatograph (XIC) and integrate the area of the peak. Peak areas were used to calculate the percent oxidation by dividing the summed areas for all forms of a given peptide in its oxidized form by the summed areas for all forms of the peptide (both reduced and oxidized) to calculate the percent oxidation. Areas from peptides with miscleavages and those detected in multiple charge states were included in the areas. A comparison of the percent oxidation between GILT-positive and GILT-negative samples was then preformed. 

### 4.13. Statistical Analysis

Data from each experimental group were subjected to statistical analysis. ANOVA with post-hoc tests, Repeated Measures ANOVA with post-hoc tests, or Student’s *t*-tests were used, as appropriate. Two-sided tests were used in all cases and *p*-values < 0.05 were considered statistically significant.

## 5. Conclusions

The data presented here suggest that the epitopes generated by GILT-expressing cells are better recognized by CD4+ T cells than those of GILT-negative cells. Enhancing the presentation of known melanoma Ags by GILT-expressing cells could be exploited through immunotherapy. Allowing the generation of in vitro T cells with specific Ags expressed by melanoma cells would also exploit immunological memory against reoccurring malignant cells.

## Data Availability

Data will be available after publication.

## Supplementary Materials

The following are available online at: https://www.mdpi.com/article/10.3390/ijms23031066/s1.

## Abbreviations

GILT	gamma-interferon-inducible lysosomal thiol reductase
HLA	human leukocyte antigen
APC	antigen-presenting cells
LC-MS/MS	liquid chromatography–tandem mass spectrometry
LC-ESI	liquid chromatography–electrospray ionization
