# Supplementary material for: GILT Expression in Human Melanoma Cells Enhances Generation of Antigenic Peptides for HLA Class II-Mediated Immune Recognition
Source: Int J Mol Sci. 2022 Jan 19;23(3):1066. doi: 10.3390/ijms23031066 (PMC8835040; doi:10.3390/ijms23031066)

Hathaway et al Supplemental Figure S1

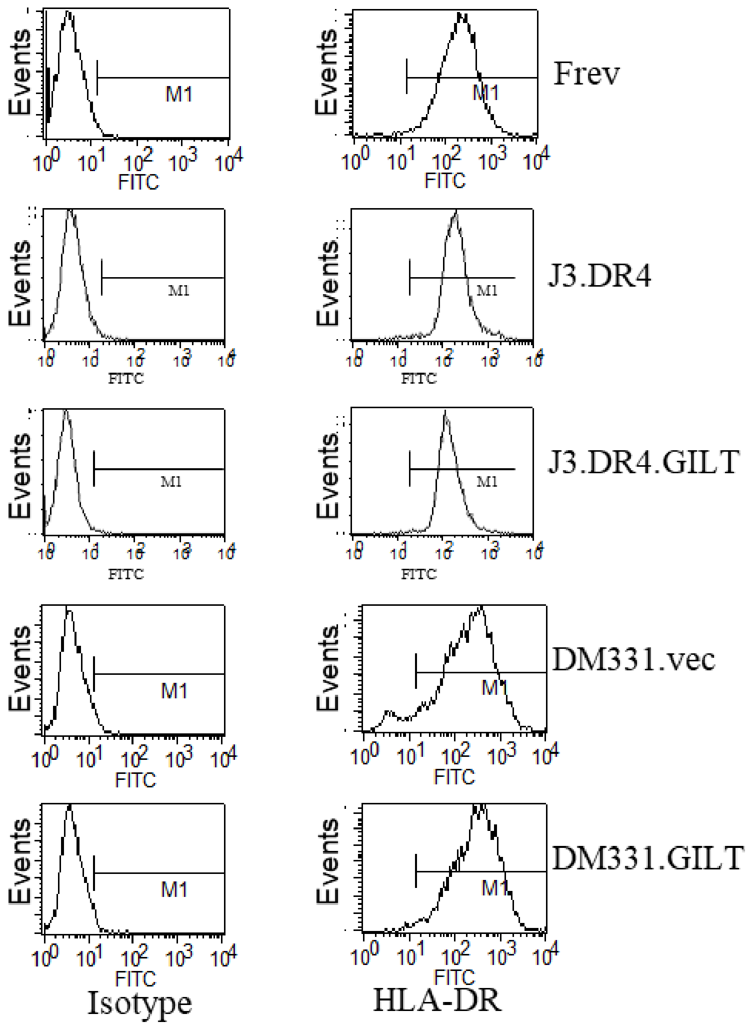

Hathaway et al Supplemental Figure S2

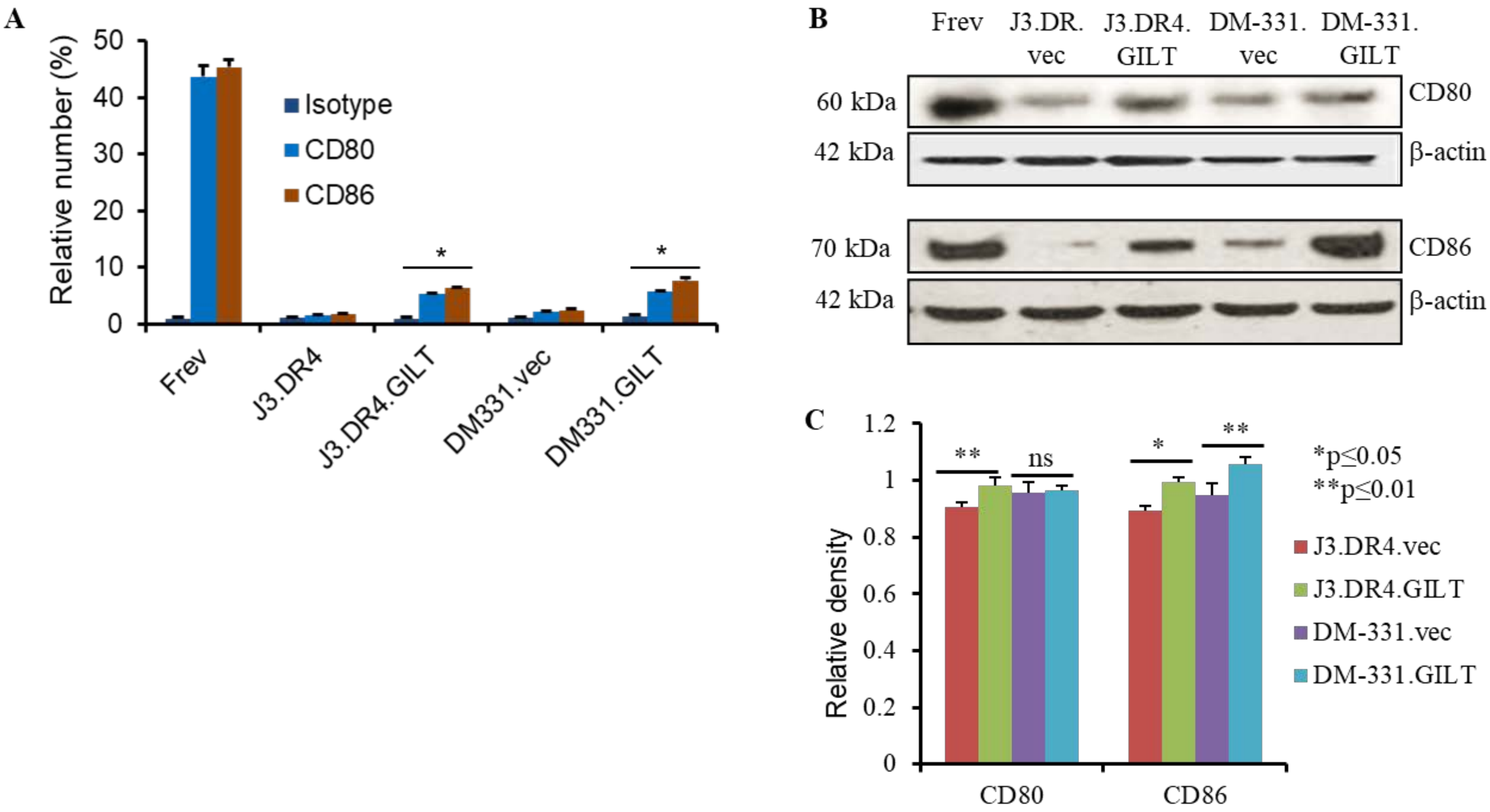

Hathaway et al Supplemental Figure S3

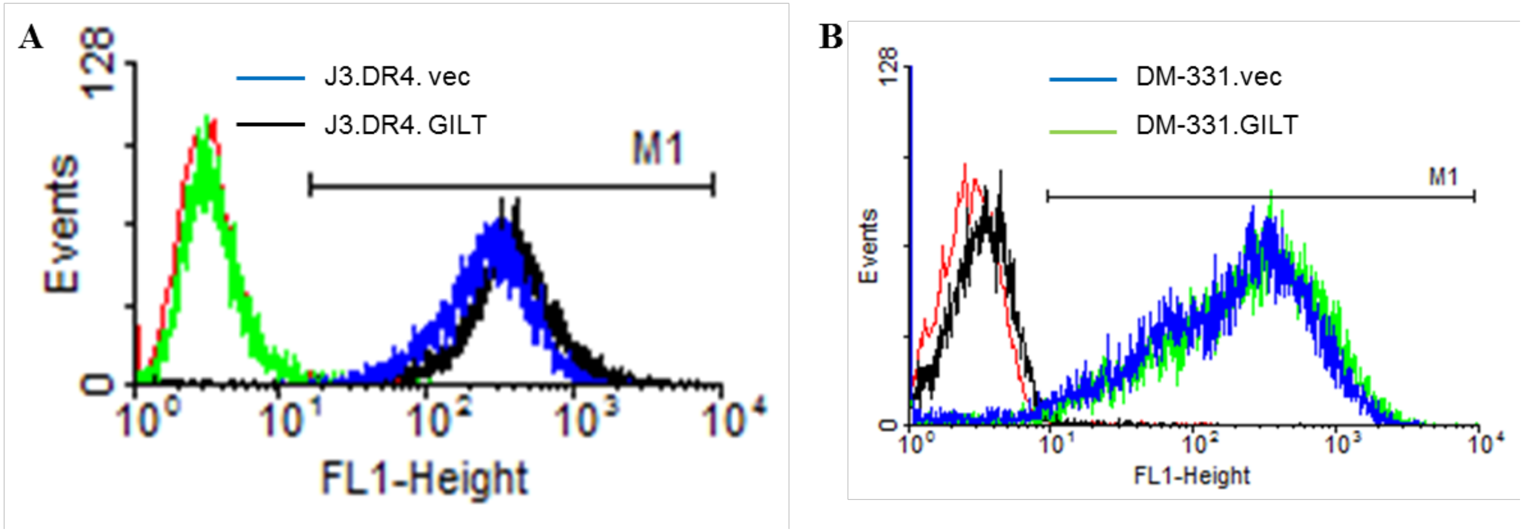

Hathaway et al Supplemental Figure S4

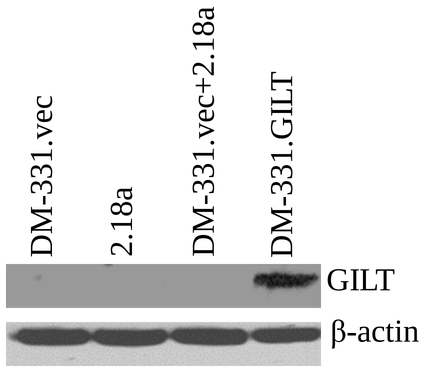

Hathaway et al Supplemental Figure S5

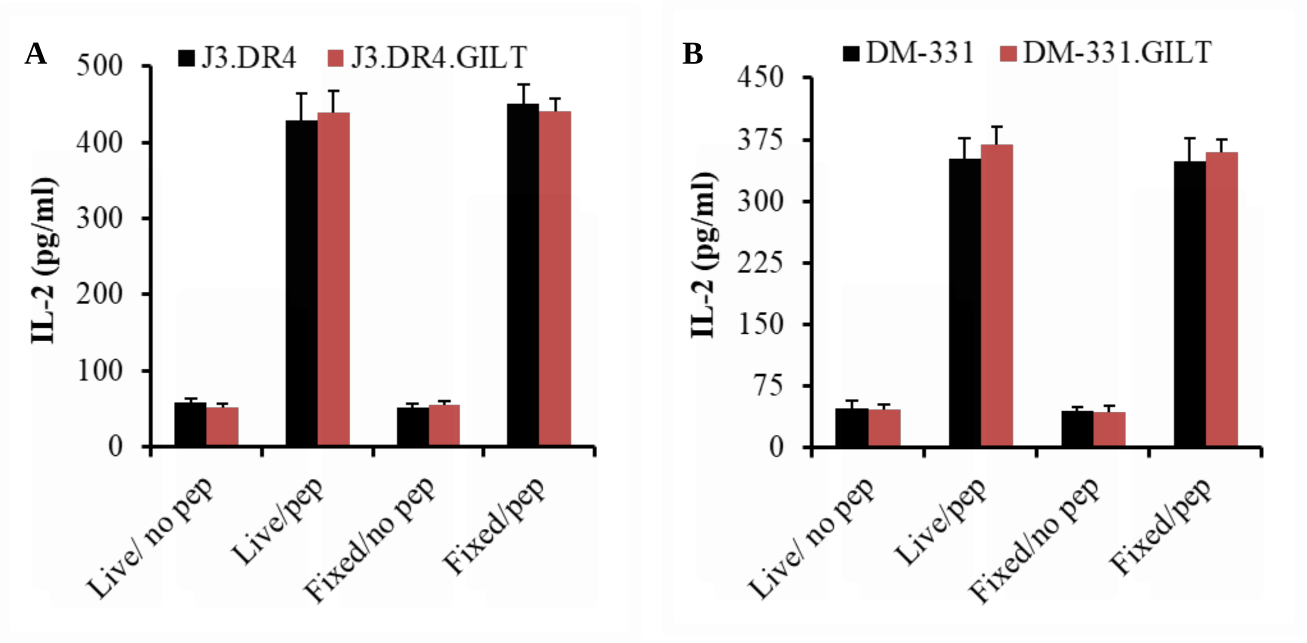

Supplement: Supplementary file 1 [file ijms-23-01066-s001.zip › ijms-1560998-supplementary.pdf]
